# 

**Published:** 1920-09-25

**Authors:** 


### New Convalescent Home at Monmouth.

The Blaina and District Workmen's Convalescent Home has been opened, in connection with the hospital at Blaina, by the purchase of Nevill Hall, Abergavenny, for many years the Monmouthshire home of the late Marquis of Abergavenny. The Home accommodates fifty patients, a number which can be increased to seventy eventually. The grounds run to thirty-three acres, and the cost of purchase and equipment amounts to £20,000, most of which has been paid.

The miners' agent for the district, Mr. James Manning, performed the opening ceremony in the presence of a large gathering, including Mr. Noah Rogers, a trustee, and one of the founders of Blaina Hospital. Mr. William Brace, M.P., spoke of the dangers of the miner's life, and said that the Home, purchased out of their pennies, had shown their power and ability for self-government.

### Wills of Medical Men.

Dr. E. N. Rycourt, of the Corner House, Ewell, Surrey, left £11,653.

Deputy Surgeon-General W. A. S. Wynne, M.D., Indian Army, of The Priory, St. Olaves, Suffolk, left £12,174.

Dr. C. D. H. Drury, of Darlington, J.P. for Durham, formerly house surgeon of the Sunderland Infirmary, and a prominent Churchman and Freemason, left estate of the value of £21,183.

### Matrons' and Sisters' Appointments.

Clare Hall Sanatorium, South Mimms, near Barnet.—Miss Wilhelmina R. Clark has been appointed sister. She was trained at the Western District Hospital, Glasgow, has been charge nurse at the Craiglochart Hospital, Edinburgh, sister at the Royal Hospital for Incurables, Edinburgh, and at the Broomhill Home for Incurables, Glasgow, and is at present doing temporary duty at the British Home and Hospital for Incurables, Streatham.

North Riding Infirmary, Middlesbrough.—Miss Clara E. Bailey has been appointed matron. She was trained at Birmingham General Hospital, where she was later second assistant matron. She has since been matron of the Jaffray Hospital, Birmingham.

General Infirmary, Peterborough.—Miss Francese E. Bellairs has been appointed night sister. She was trained at Halifax Royal Infirmary, and has since been sister at Newark General Hospital, at Halifax Royal Infirmary, and under the British Red Cross Society.

Southmead Infirmary, Bristol.—Miss Elizabeth Oude has been appointed home sister. She was trained at Eastville Infirmary, Bristol, where she was later charge nurse. She has since been at Bath War Hospital, and later returned to Eastville.

St. George's Home, Milman's Street, Chelsea.—Miss Jean Wallace Denchar has been appointed sister. She was trained at Liverpool Royal Infirmary and at Ruchill Fever Hospital, Glasgow, and has since been sister at Parkhill Hospital, Liverpool, at York County Hospital, and at Monsall Fever Hospital, Manchester, and out-patient sister and housekeeping sister at Liverpool Royal Infirmary.

Berwick-on-Tweed Infirmary.—Miss Davida M. F. Forsyth has been appointed matron. She was trained at the Victoria Infirmary, Glasgow, has been a member of the staff of the Edinburgh War Hospital, Bangour, West Lothian, of a nursing home in Glasgow, and has done private nursing in Edinburgh.

St. Luke's Hospital, Halifax.—Miss Annie G. Angus has been appointed sister of the paying maternity department. She was trained at the Mill Road Infirmary, Liverpool, and received her midwifery training at the Liverpool Maternity Hospital, where she has since been sister.

Royal Infirmary, Gloucester.—Miss B. Rees and Miss T. Cessford have been appointed out-patient and casualty sister and ward sister respectively. Miss Rees was trained at the Royal Infirmary, Bradford, and has held posts at the Winchmore Hill War Hospital and Dundee War Hospital. Miss Cessford was trained at the Royal Infirmary, Sheffield, and has since been sister at the Beckett Hospital, Barnsley, and at the Borough Sanatorium, Hastings.

### Aberystwyth Infirmary Extension.

Gratifying success has attended the effort to raise £10,000 to enable the Aberystwyth Infirmary and Cardiganshire General Hospital to extend its buildings. Contingent on a similar sum being obtained locally, £3,000 was offered by the Red Cross Society. The leading feature of the week's effort, which was organised by a committee under Dr. Roberts, was a three-days' bazaar at the University College, Aberystwyth, which, with the private gifts handed in, realised £6,500. A presentation of purses totalled £1,200, and the ex-Service men of the county generously sent a cheque for £600, being their share of the "Byng Millions," as they are popularly called. Other contributions helping to swell the total were £80 from a local Sunday School and £120 from the Earl of Lisburne, whose wife opened the bazaar on the initial day.

